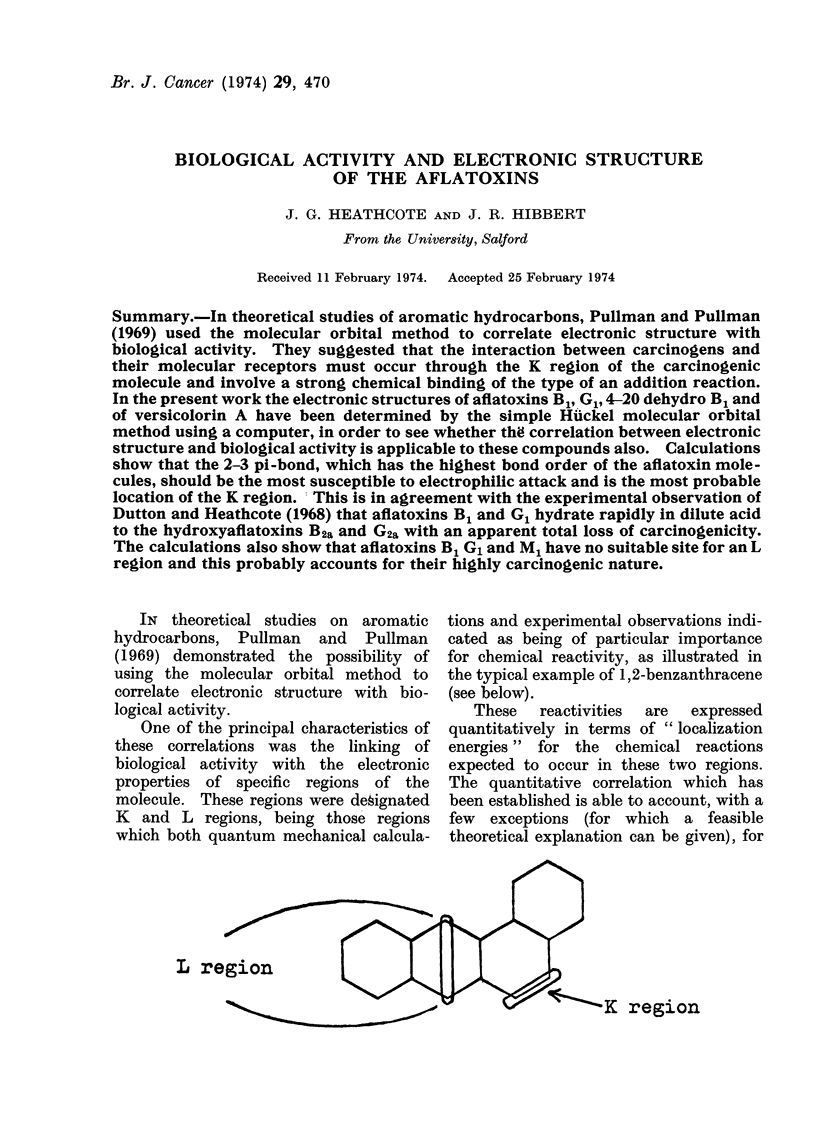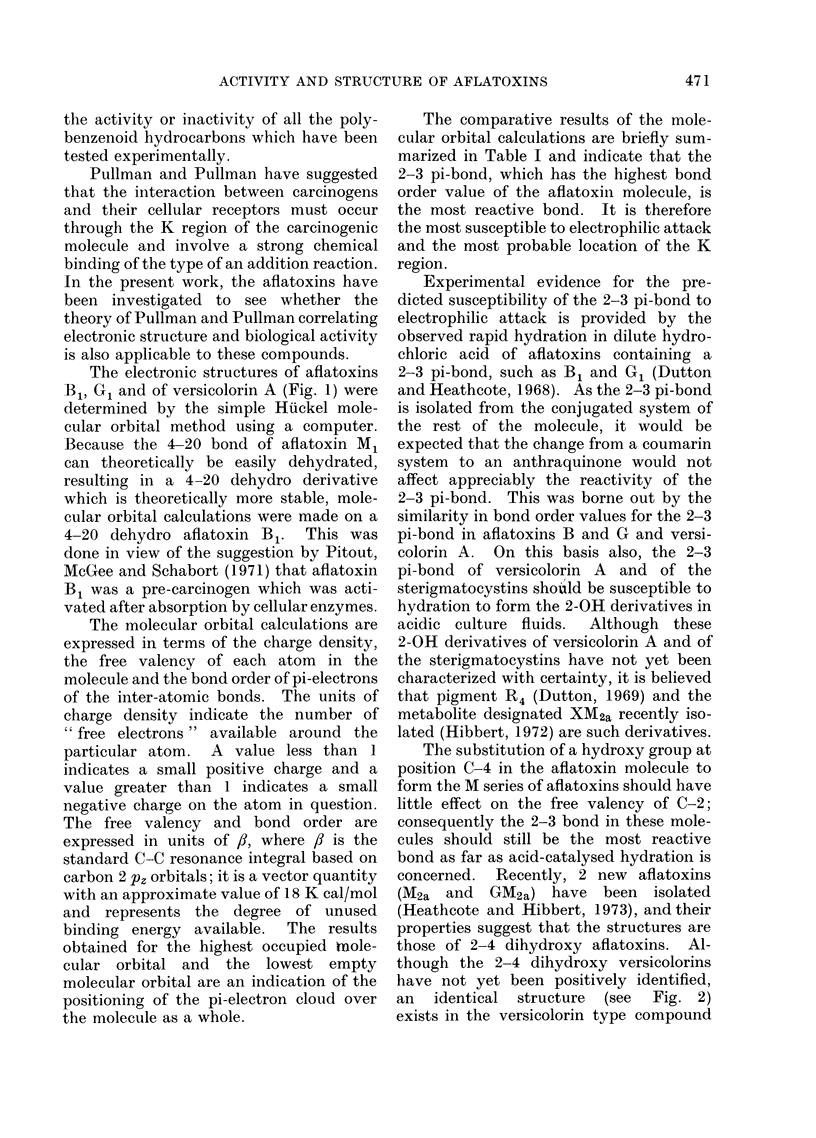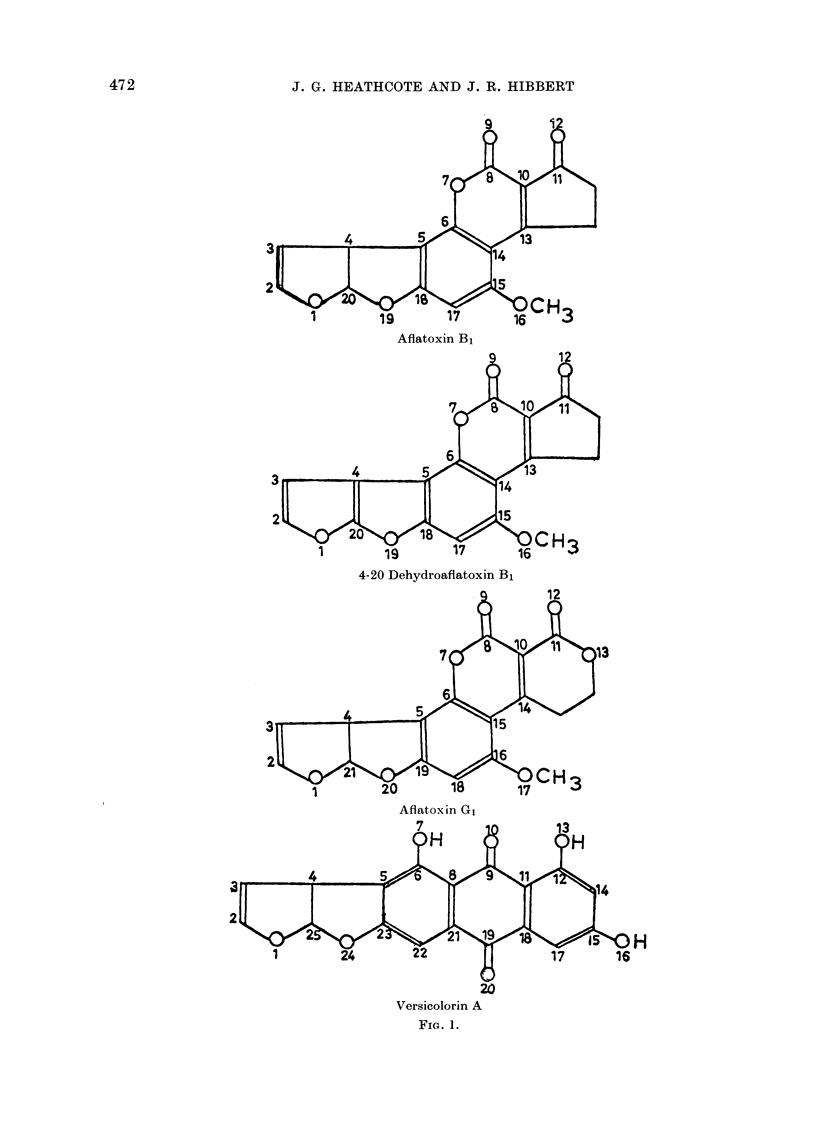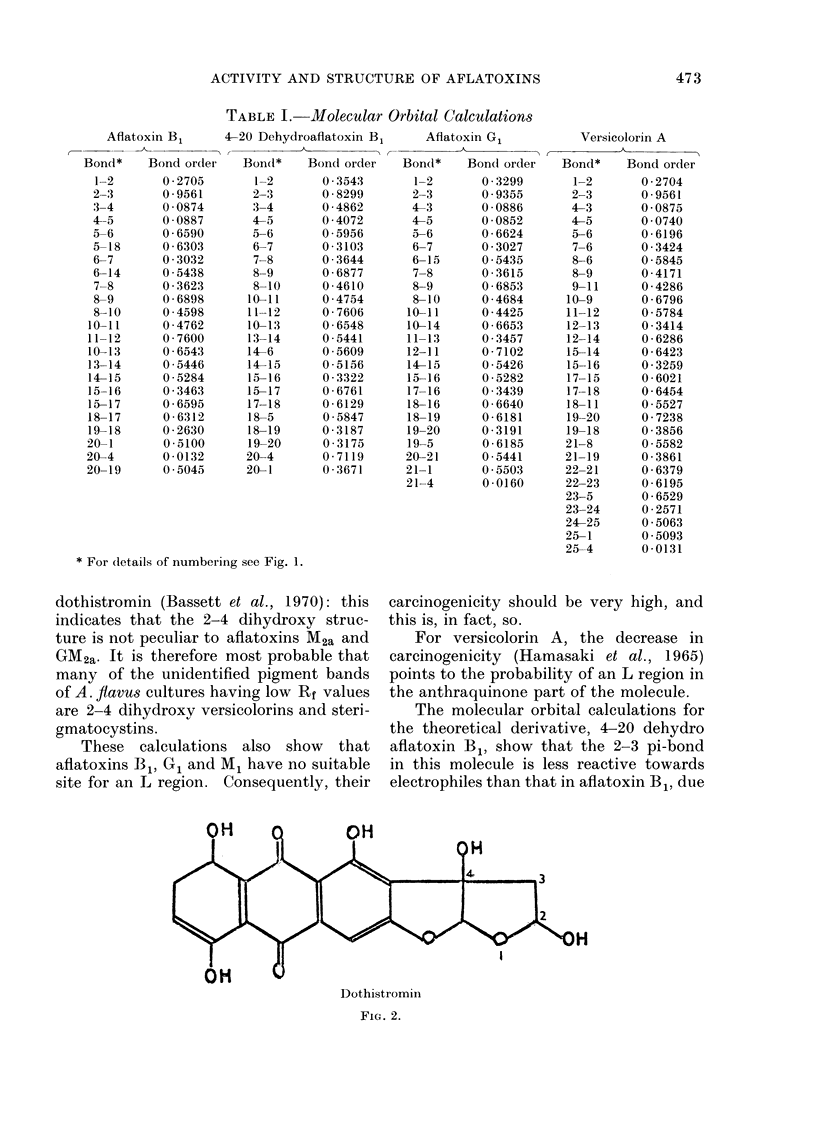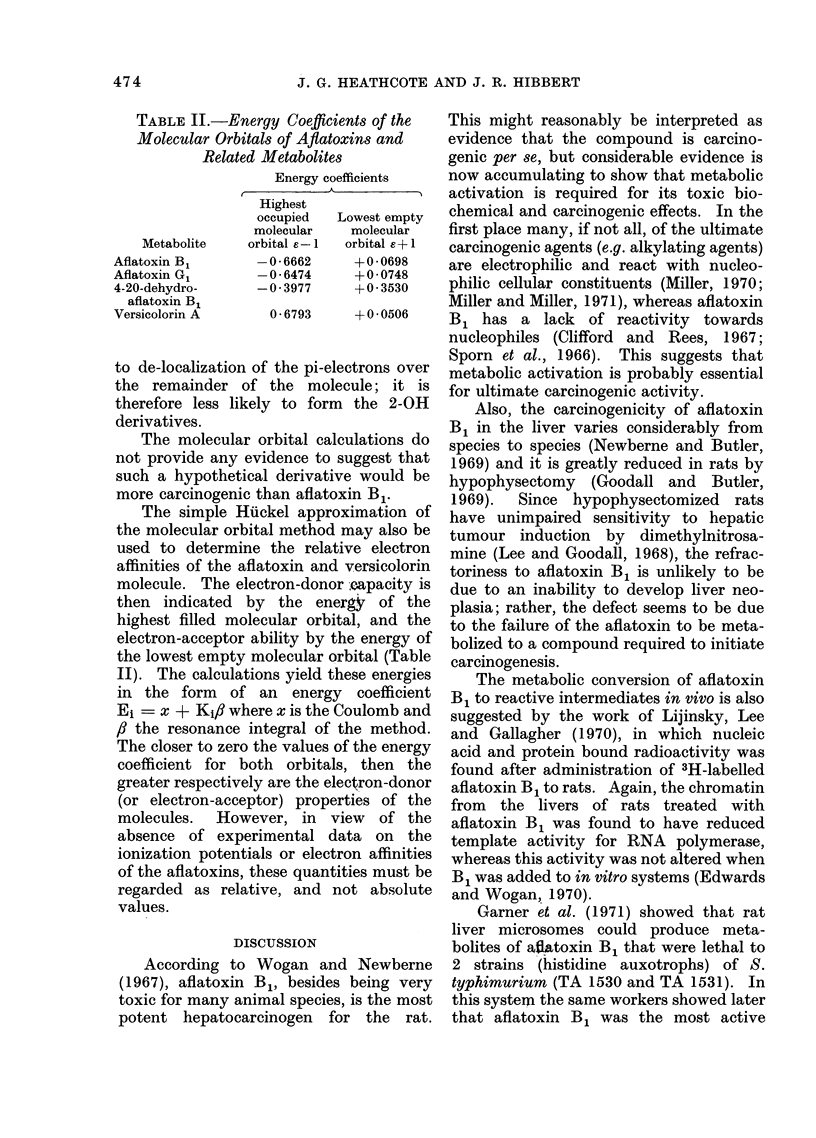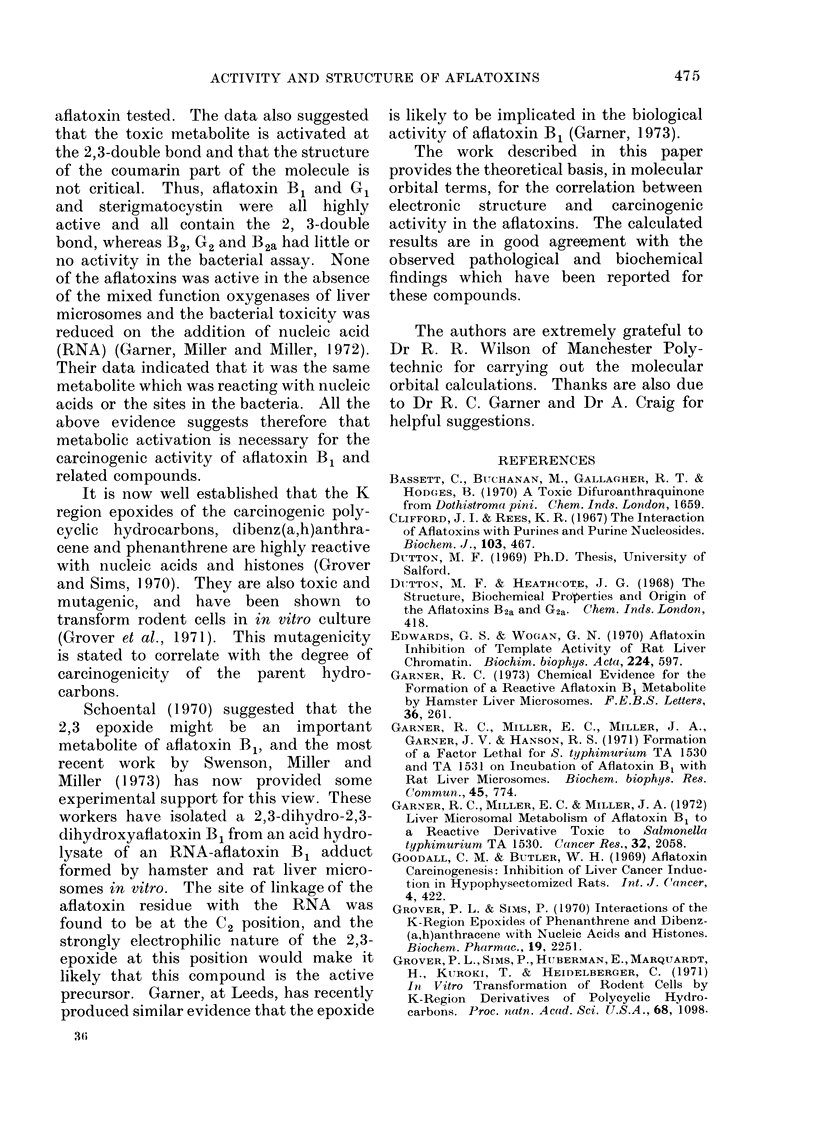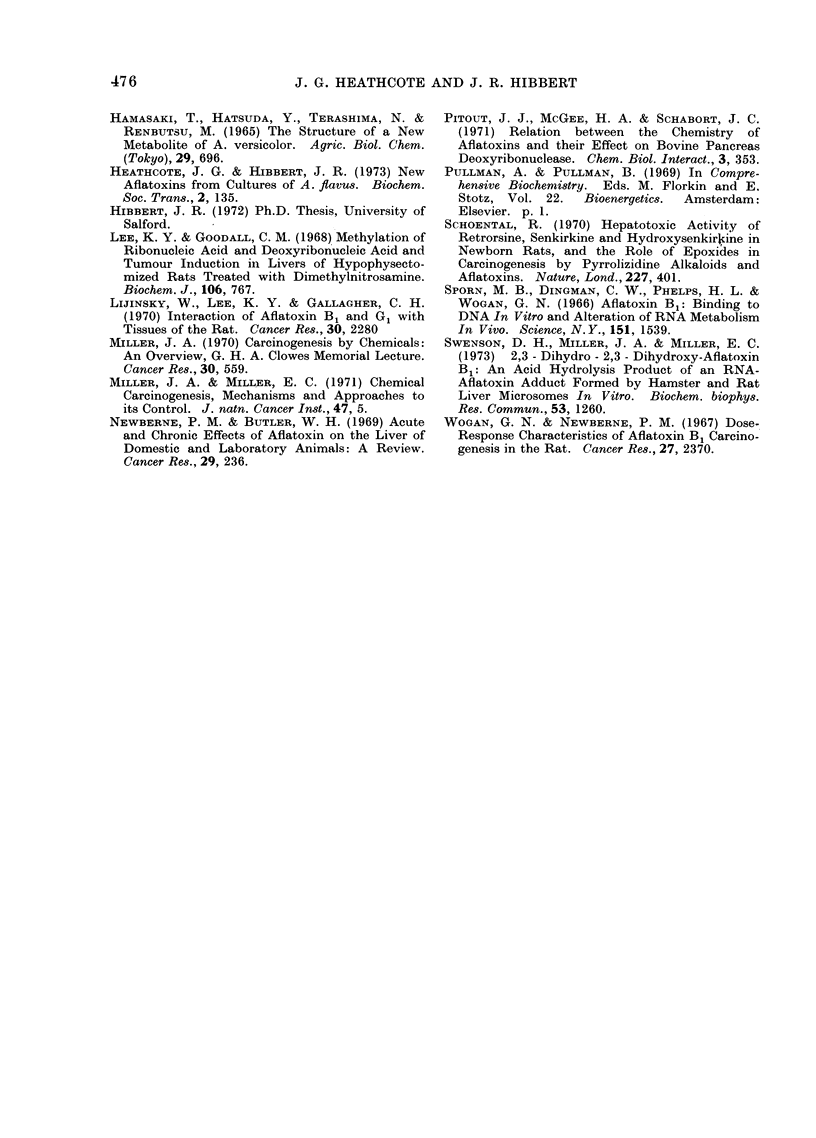# Biological Activity and Electronic Structure of the Aflatoxins

**DOI:** 10.1038/bjc.1974.99

**Published:** 1974-06

**Authors:** J. G. Heathcote, J. R. Hibbert

## Abstract

In theoretical studies of aromatic hydrocarbons, Pullman and Pullman (1969) used the molecular orbital method to correlate electronic structure with biological activity. They suggested that the interaction between carcinogens and their molecular receptors must occur through the K region of the carcinogenic molecule and involve a strong chemical binding of the type of an addition reaction. In the present work the electronic structures of aflatoxins B_1_, G_1_, 4-20 dehydro B_1_ and of versicolorin A have been determined by the simple Hückel molecular orbital method using a computer, in order to see whether the correlation between electronic structure and biological activity is applicable to these compounds also. Calculations show that the 2-3 pi-bond, which has the highest bond order of the aflatoxin molecules, should be the most susceptible to electrophilic attack and is the most probable location of the K region. This is in agreement with the experimental observation of Dutton and Heathcote (1968) that aflatoxins B_1_ and G_1_ hydrate rapidly in dilute acid to the hydroxyaflatoxins B_2a_ and G_2a_ with an apparent total loss of carcinogenicity. The calculations also show that aflatoxins B_1_ G_1_ and M_1_ have no suitable site for an L region and this probably accounts for their highly carcinogenic nature.


*Br. J. Cancer* (1974) **29**, 470

### BIOLOGICAL ACTIVITY AND ELECTRONIC STRUCTURE OF THE AFLATOXINS

J. G. HEATHCOTE AND J. R. HIBBERT

*From the University, Salford*

Received 11 February 1974. Accepted 25 February 1974

**Summary.—In theoretical studies of aromatic hydrocarbons, Pullman and Pullman (1969) used the molecular orbital method to correlate electronic structure with biological activity. They suggested that the interaction between carcinogens and their molecular receptors must occur through the K region of the carcinogenic molecule and involve a strong chemical binding of the type of an addition reaction. In the present work the electronic structures of aflatoxins $B_1$, $G_1$, 4–20 dehydro $B_1$ and of versicolorin A have been determined by the simple Hückel molecular orbital method using a computer, in order to see whether the correlation between electronic structure and biological activity is applicable to these compounds also. Calculations show that the 2–3 pi-bond, which has the highest bond order of the aflatoxin molecules, should be the most susceptible to electrophilic attack and is the most probable location of the K region. This is in agreement with the experimental observation of Dutton and Heathcote (1968) that aflatoxins $B_1$ and $G_1$ hydrate rapidly in dilute acid to the hydroxyaflatoxins $B_{2a}$ and $G_{2a}$ with an apparent total loss of carcinogenicity. The calculations also show that aflatoxins $B_1$ $G_1$ and $M_1$ have no suitable site for an L region and this probably accounts for their highly carcinogenic nature.**

In theoretical studies on aromatic hydrocarbons, Pullman and Pullman (1969) demonstrated the possibility of using the molecular orbital method to correlate electronic structure with biological activity.

One of the principal characteristics of these correlations was the linking of biological activity with the electronic properties of specific regions of the molecule. These regions were designated K and L regions, being those regions which both quantum mechanical calculations and experimental observations indicated as being of particular importance for chemical reactivity, as illustrated in the typical example of 1,2-benzanthracene (see below).

These reactivities are expressed quantitatively in terms of "localization energies" for the chemical reactions expected to occur in these two regions. The quantitative correlation which has been established is able to account, with a few exceptions (for which a feasible theoretical explanation can be given), for the activity or inactivity of all the polybenzenoid hydrocarbons which have been tested experimentally.

Pullman and Pullman have suggested that the interaction between carcinogens and their cellular receptors must occur through the K region of the carcinogenic molecule and involve a strong chemical binding of the type of an addition reaction. In the present work, the aflatoxins have been investigated to see whether the theory of Pullman and Pullman correlating electronic structure and biological activity is also applicable to these compounds.

The electronic structures of aflatoxins $B_1$, $G_1$ and of versicolorin A (Fig. 1) were determined by the simple Hückel molecular orbital method using a computer. Because the 4–20 bond of aflatoxin $M_1$ can theoretically be easily dehydrated, resulting in a 4–20 dehydro derivative which is theoretically more stable, molecular orbital calculations were made on a 4–20 dehydro aflatoxin $B_1$. This was done in view of the suggestion by Pitout, McGee and Schabort (1971) that aflatoxin $B_1$ was a pre-carcinogen which was activated after absorption by cellular enzymes.

The molecular orbital calculations are expressed in terms of the charge density, the free valency of each atom in the molecule and the bond order of pi-electrons of the inter-atomic bonds. The units of charge density indicate the number of "free electrons" available around the particular atom. A value less than 1 indicates a small positive charge and a value greater than 1 indicates a small negative charge on the atom in question. The free valency and bond order are expressed in units of $\beta$, where $\beta$ is the standard C–C resonance integral based on carbon 2 $p_z$ orbitals; it is a vector quantity with an approximate value of 18 K cal/mol and represents the degree of unused binding energy available. The results obtained for the highest occupied molecular orbital and the lowest empty molecular orbital are an indication of the positioning of the pi-electron cloud over the molecule as a whole.

The comparative results of the molecular orbital calculations are briefly summarized in Table I and indicate that the 2–3 pi-bond, which has the highest bond order value of the aflatoxin molecule, is the most reactive bond. It is therefore the most susceptible to electrophilic attack and the most probable location of the K region.

Experimental evidence for the predicted susceptibility of the 2–3 pi-bond to electrophilic attack is provided by the observed rapid hydration in dilute hydrochloric acid of aflatoxins containing a 2–3 pi-bond, such as $B_1$ and $G_1$ (Dutton and Heathcote, 1968). As the 2–3 pi-bond is isolated from the conjugated system of the rest of the molecule, it would be expected that the change from a coumarin system to an anthraquinone would not affect appreciably the reactivity of the 2–3 pi-bond. This was borne out by the similarity in bond order values for the 2–3 pi-bond in aflatoxins B and G and versicolorin A. On this basis also, the 2–3 pi-bond of versicolorin A and of the sterigmatocystins should be susceptible to hydration to form the 2-OH derivatives in acidic culture fluids. Although these 2-OH derivatives of versicolorin A and of the sterigmatocystins have not yet been characterized with certainty, it is believed that pigment $R_4$ (Dutton, 1969) and the metabolite designated $XM_{2a}$ recently isolated (Hibbert, 1972) are such derivatives.

The substitution of a hydroxy group at position C–4 in the aflatoxin molecule to form the M series of aflatoxins should have little effect on the free valency of C–2; consequently the 2–3 bond in these molecules should still be the most reactive bond as far as acid-catalysed hydration is concerned. Recently, 2 new aflatoxins ($M_{2a}$ and $GM_{2a}$) have been isolated (Heathcote and Hibbert, 1973), and their properties suggest that the structures are those of 2–4 dihydroxy aflatoxins. Although the 2–4 dihydroxy versicolorins have not yet been positively identified, an identical structure (see Fig. 2) exists in the versicolorin type compound

Aflatoxin $B_1$

4-20 Dehydroaflatoxin $B_1$

Aflatoxin $G_1$

Versicolorin A

FIG. 1.

TABLE I.—*Molecular Orbital Calculations*

| Aflatoxin $B_1$ | | 4–20 Dehydroaflatoxin $B_1$ | | Aflatoxin $G_1$ | | Versicolorin A | |
|---|---|---|---|---|---|---|---|
| Bond* | Bond order | Bond* | Bond order | Bond* | Bond order | Bond* | Bond order |
| 1–2 | 0·2705 | 1–2 | 0·3543 | 1–2 | 0·3299 | 1–2 | 0·2704 |
| 2–3 | 0·9561 | 2–3 | 0·8299 | 2–3 | 0·9355 | 2–3 | 0·9561 |
| 3–4 | 0·0874 | 3–4 | 0·4862 | 4–3 | 0·0886 | 4–3 | 0·0875 |
| 4–5 | 0·0887 | 4–5 | 0·4072 | 4–5 | 0·0852 | 4–5 | 0·0740 |
| 5–6 | 0·6590 | 5–6 | 0·5956 | 5–6 | 0·6624 | 5–6 | 0·6196 |
| 5–18 | 0·6303 | 6–7 | 0·3103 | 6–7 | 0·3027 | 7–6 | 0·3424 |
| 6–7 | 0·3032 | 7–8 | 0·3644 | 6–15 | 0·5435 | 8–6 | 0·5845 |
| 6–14 | 0·5438 | 8–9 | 0·6877 | 7–8 | 0·3615 | 8–9 | 0·4171 |
| 7–8 | 0·3623 | 8–10 | 0·4610 | 8–9 | 0·6853 | 9–11 | 0·4286 |
| 8–9 | 0·6898 | 10–11 | 0·4754 | 8–10 | 0·4684 | 10–9 | 0·6796 |
| 8–10 | 0·4598 | 11–12 | 0·7606 | 10–11 | 0·4425 | 11–12 | 0·5784 |
| 10–11 | 0·4762 | 10–13 | 0·6548 | 10–14 | 0·6653 | 12–13 | 0·3414 |
| 11–12 | 0·7600 | 13–14 | 0·5441 | 11–13 | 0·3457 | 12–14 | 0·6286 |
| 10–13 | 0·6543 | 14–6 | 0·5609 | 12–11 | 0·7102 | 15–14 | 0·6423 |
| 13–14 | 0·5446 | 14–15 | 0·5156 | 14–15 | 0·5426 | 15–16 | 0·3259 |
| 14–15 | 0·5284 | 15–16 | 0·3322 | 15–16 | 0·5282 | 17–15 | 0·6021 |
| 15–16 | 0·3463 | 15–17 | 0·6761 | 17–16 | 0·3439 | 17–18 | 0·6454 |
| 15–17 | 0·6595 | 17–18 | 0·6129 | 18–16 | 0·6640 | 18–11 | 0·5527 |
| 18–17 | 0·6312 | 18–5 | 0·5847 | 18–19 | 0·6181 | 19–20 | 0·7238 |
| 19–18 | 0·2630 | 18–19 | 0·3187 | 19–20 | 0·3191 | 19–18 | 0·3856 |
| 20–1 | 0·5100 | 19–20 | 0·3175 | 19–5 | 0·6185 | 21–8 | 0·5582 |
| 20–4 | 0·0132 | 20–4 | 0·7119 | 20–21 | 0·5441 | 21–19 | 0·3861 |
| 20–19 | 0·5045 | 20–1 | 0·3671 | 21–1 | 0·5503 | 22–21 | 0·6379 |
| | | | | 21–4 | 0·0160 | 22–23 | 0·6195 |
| | | | | | | 23–5 | 0·6529 |
| | | | | | | 23–24 | 0·2571 |
| | | | | | | 24–25 | 0·5063 |
| | | | | | | 25–1 | 0·5093 |
| | | | | | | 25–4 | 0·0131 |

* For details of numbering see Fig. 1.

dothistromin (Bassett *et al.*, 1970): this indicates that the 2–4 dihydroxy structure is not peculiar to aflatoxins $M_{2a}$ and $GM_{2a}$. It is therefore most probable that many of the unidentified pigment bands of *A. flavus* cultures having low $R_f$ values are 2–4 dihydroxy versicolorins and sterigmatocystins.

These calculations also show that aflatoxins $B_1$, $G_1$ and $M_1$ have no suitable site for an L region. Consequently, their carcinogenicity should be very high, and this is, in fact, so.

For versicolorin A, the decrease in carcinogenicity (Hamasaki *et al.*, 1965) points to the probability of an L region in the anthraquinone part of the molecule.

The molecular orbital calculations for the theoretical derivative, 4–20 dehydro aflatoxin $B_1$, show that the 2–3 pi-bond in this molecule is less reactive towards electrophiles than that in aflatoxin $B_1$, due

Dothistromin

FIG. 2.

TABLE II.—*Energy Coefficients of the Molecular Orbitals of Aflatoxins and Related Metabolites*

| Metabolite | Energy coefficients: Highest occupied molecular orbital $\varepsilon - 1$ | Lowest empty molecular orbital $\varepsilon + 1$ |
|---|---|---|
| Aflatoxin $B_1$ | $-0 \cdot 6662$ | $+0 \cdot 0698$ |
| Aflatoxin $G_1$ | $-0 \cdot 6474$ | $+0 \cdot 0748$ |
| 4-20-dehydro-aflatoxin $B_1$ | $-0 \cdot 3977$ | $+0 \cdot 3530$ |
| Versicolorin A | $0 \cdot 6793$ | $+0 \cdot 0506$ |

to de-localization of the pi-electrons over the remainder of the molecule; it is therefore less likely to form the 2-OH derivatives.

The molecular orbital calculations do not provide any evidence to suggest that such a hypothetical derivative would be more carcinogenic than aflatoxin $B_1$.

The simple Hückel approximation of the molecular orbital method may also be used to determine the relative electron affinities of the aflatoxin and versicolorin molecule. The electron-donor capacity is then indicated by the energy of the highest filled molecular orbital, and the electron-acceptor ability by the energy of the lowest empty molecular orbital (Table II). The calculations yield these energies in the form of an energy coefficient $E_i = x + K_i\beta$ where $x$ is the Coulomb and $\beta$ the resonance integral of the method. The closer to zero the values of the energy coefficient for both orbitals, then the greater respectively are the electron-donor (or electron-acceptor) properties of the molecules. However, in view of the absence of experimental data on the ionization potentials or electron affinities of the aflatoxins, these quantities must be regarded as relative, and not absolute values.

### DISCUSSION

According to Wogan and Newberne (1967), aflatoxin $B_1$, besides being very toxic for many animal species, is the most potent hepatocarcinogen for the rat. This might reasonably be interpreted as evidence that the compound is carcinogenic *per se*, but considerable evidence is now accumulating to show that metabolic activation is required for its toxic biochemical and carcinogenic effects. In the first place many, if not all, of the ultimate carcinogenic agents (*e.g.* alkylating agents) are electrophilic and react with nucleophilic cellular constituents (Miller, 1970; Miller and Miller, 1971), whereas aflatoxin $B_1$ has a lack of reactivity towards nucleophiles (Clifford and Rees, 1967; Sporn *et al.*, 1966). This suggests that metabolic activation is probably essential for ultimate carcinogenic activity.

Also, the carcinogenicity of aflatoxin $B_1$ in the liver varies considerably from species to species (Newberne and Butler, 1969) and it is greatly reduced in rats by hypophysectomy (Goodall and Butler, 1969). Since hypophysectomized rats have unimpaired sensitivity to hepatic tumour induction by dimethylnitrosamine (Lee and Goodall, 1968), the refractoriness to aflatoxin $B_1$ is unlikely to be due to an inability to develop liver neoplasia; rather, the defect seems to be due to the failure of the aflatoxin to be metabolized to a compound required to initiate carcinogenesis.

The metabolic conversion of aflatoxin $B_1$ to reactive intermediates *in vivo* is also suggested by the work of Lijinsky, Lee and Gallagher (1970), in which nucleic acid and protein bound radioactivity was found after administration of $^3H$-labelled aflatoxin $B_1$ to rats. Again, the chromatin from the livers of rats treated with aflatoxin $B_1$ was found to have reduced template activity for RNA polymerase, whereas this activity was not altered when $B_1$ was added to *in vitro* systems (Edwards and Wogan, 1970).

Garner *et al.* (1971) showed that rat liver microsomes could produce metabolites of aflatoxin $B_1$ that were lethal to 2 strains (histidine auxotrophs) of *S. typhimurium* (TA 1530 and TA 1531). In this system the same workers showed later that aflatoxin $B_1$ was the most active aflatoxin tested. The data also suggested that the toxic metabolite is activated at the 2,3-double bond and that the structure of the coumarin part of the molecule is not critical. Thus, aflatoxin $B_1$ and $G_1$ and sterigmatocystin were all highly active and all contain the 2, 3-double bond, whereas $B_2$, $G_2$ and $B_{2a}$ had little or no activity in the bacterial assay. None of the aflatoxins was active in the absence of the mixed function oxygenases of liver microsomes and the bacterial toxicity was reduced on the addition of nucleic acid (RNA) (Garner, Miller and Miller, 1972). Their data indicated that it was the same metabolite which was reacting with nucleic acids or the sites in the bacteria. All the above evidence suggests therefore that metabolic activation is necessary for the carcinogenic activity of aflatoxin $B_1$ and related compounds.

It is now well established that the K region epoxides of the carcinogenic polycyclic hydrocarbons, dibenz(a,h)anthracene and phenanthrene are highly reactive with nucleic acids and histones (Grover and Sims, 1970). They are also toxic and mutagenic, and have been shown to transform rodent cells in *in vitro* culture (Grover *et al.*, 1971). This mutagenicity is stated to correlate with the degree of carcinogenicity of the parent hydrocarbons.

Schoental (1970) suggested that the 2,3 epoxide might be an important metabolite of aflatoxin $B_1$, and the most recent work by Swenson, Miller and Miller (1973) has now provided some experimental support for this view. These workers have isolated a 2,3-dihydro-2,3-dihydroxyaflatoxin $B_1$ from an acid hydrolysate of an RNA-aflatoxin $B_1$ adduct formed by hamster and rat liver microsomes *in vitro*. The site of linkage of the aflatoxin residue with the RNA was found to be at the $C_2$ position, and the strongly electrophilic nature of the 2,3-epoxide at this position would make it likely that this compound is the active precursor. Garner, at Leeds, has recently produced similar evidence that the epoxide is likely to be implicated in the biological activity of aflatoxin $B_1$ (Garner, 1973).

The work described in this paper provides the theoretical basis, in molecular orbital terms, for the correlation between electronic structure and carcinogenic activity in the aflatoxins. The calculated results are in good agreement with the observed pathological and biochemical findings which have been reported for these compounds.

The authors are extremely grateful to Dr R. R. Wilson of Manchester Polytechnic for carrying out the molecular orbital calculations. Thanks are also due to Dr R. C. Garner and Dr A. Craig for helpful suggestions.

### REFERENCES

Bassett, C., Buchanan, M., Gallagher, R. T. & Hodges, B. (1970) A Toxic Difuroanthraquinone from *Dothistroma pini*. *Chem. Inds. London*, 1659.

Clifford, J. I. & Rees, K. R. (1967) The Interaction of Aflatoxins with Purines and Purine Nucleosides. *Biochem. J.*, **103**, 467.

Dutton, M. F. (1969) Ph.D. Thesis, University of Salford.

Dutton, M. F. & Heathcote, J. G. (1968) The Structure, Biochemical Properties and Origin of the Aflatoxins $B_{2a}$ and $G_{2a}$. *Chem. Inds. London*, 418.

Edwards, G. S. & Wogan, G. N. (1970) Aflatoxin Inhibition of Template Activity of Rat Liver Chromatin. *Biochim. biophys. Acta*, **224**, 597.

Garner, R. C. (1973) Chemical Evidence for the Formation of a Reactive Aflatoxin $B_1$ Metabolite by Hamster Liver Microsomes. *F.E.B.S. Letters*, **36**, 261.

Garner, R. C., Miller, E. C., Miller, J. A., Garner, J. V. & Hanson, R. S. (1971) Formation of a Factor Lethal for *S. typhimurium* TA 1530 and TA 1531 on Incubation of Aflatoxin $B_1$ with Rat Liver Microsomes. *Biochem. biophys. Res. Commun.*, **45**, 774.

Garner, R. C., Miller, E. C. & Miller, J. A. (1972) Liver Microsomal Metabolism of Aflatoxin $B_1$ to a Reactive Derivative Toxic to *Salmonella typhimurium* TA 1530. *Cancer Res.*, **32**, 2058.

Goodall, C. M. & Butler, W. H. (1969) Aflatoxin Carcinogenesis: Inhibition of Liver Cancer Induction in Hypophysectomized Rats. *Int. J. Cancer*, **4**, 422.

Grover, P. L. & Sims, P. (1970) Interactions of the K-Region Epoxides of Phenanthrene and Dibenz-(a,h)anthracene with Nucleic Acids and Histones. *Biochem. Pharmac.*, **19**, 2251.

Grover, P. L., Sims, P., Huberman, E., Marquardt, H., Kuroki, T. & Heidelberger, C. (1971) *In Vitro* Transformation of Rodent Cells by K-Region Derivatives of Polycyclic Hydrocarbons. *Proc. natn. Acad. Sci. U.S.A.*, **68**, 1098.

HAMASAKI, T., HATSUDA, Y., TERASHIMA, N. & RENBUTSU, M. (1965) The Structure of a New Metabolite of A. versicolor. *Agric. Biol. Chem. (Tokyo)*, **29**, 696.

HEATHCOTE, J. G. & HIBBERT, J. R. (1973) New Aflatoxins from Cultures of *A. flavus*. *Biochem. Soc. Trans.*, **2**, 135.

HIBBERT, J. R. (1972) Ph.D. Thesis, University of Salford.

LEE, K. Y. & GOODALL, C. M. (1968) Methylation of Ribonucleic Acid and Deoxyribonucleic Acid and Tumour Induction in Livers of Hypophysectomized Rats Treated with Dimethylnitrosamine. *Biochem. J.*, **106**, 767.

LIJINSKY, W., LEE, K. Y. & GALLAGHER, C. H. (1970) Interaction of Aflatoxin $B_1$ and $G_1$ with Tissues of the Rat. *Cancer Res.*, **30**, 2280

MILLER, J. A. (1970) Carcinogenesis by Chemicals: An Overview, G. H. A. Clowes Memorial Lecture. *Cancer Res.*, **30**, 559.

MILLER, J. A. & MILLER, E. C. (1971) Chemical Carcinogenesis, Mechanisms and Approaches to its Control. *J. natn. Cancer Inst.*, **47**, 5.

NEWBERNE, P. M. & BUTLER, W. H. (1969) Acute and Chronic Effects of Aflatoxin on the Liver of Domestic and Laboratory Animals: A Review. *Cancer Res.*, **29**, 236.

PITOUT, J. J., MCGEE, H. A. & SCHABORT, J. C. (1971) Relation between the Chemistry of Aflatoxins and their Effect on Bovine Pancreas Deoxyribonuclease. *Chem. Biol. Interact.*, **3**, 353.

PULLMAN, A. & PULLMAN, B. (1969) In *Comprehensive Biochemistry*. Eds. M. Florkin and E. Stotz, Vol. 22. *Bioenergetics*. Amsterdam: Elsevier. p. 1.

SCHOENTAL, R. (1970) Hepatotoxic Activity of Retrorsine, Senkirkine and Hydroxysenkirkine in Newborn Rats, and the Role of Epoxides in Carcinogenesis by Pyrrolizidine Alkaloids and Aflatoxins. *Nature, Lond.*, **227**, 401.

SPORN, M. B., DINGMAN, C. W., PHELPS, H. L. & WOGAN, G. N. (1966) Aflatoxin $B_1$: Binding to DNA *In Vitro* and Alteration of RNA Metabolism *In Vivo*. *Science, N.Y.*, **151**, 1539.

SWENSON, D. H., MILLER, J. A. & MILLER, E. C. (1973) 2,3 - Dihydro - 2,3 - Dihydroxy-Aflatoxin $B_1$: An Acid Hydrolysis Product of an RNA-Aflatoxin Adduct Formed by Hamster and Rat Liver Microsomes *In Vitro*. *Biochem. biophys. Res. Commun.*, **53**, 1260.

WOGAN, G. N. & NEWBERNE, P. M. (1967) Dose-Response Characteristics of Aflatoxin $B_1$ Carcinogenesis in the Rat. *Cancer Res.*, **27**, 2370.